# Value of carotid corrected flow time or changes value of FTc could be more useful in predicting fluid responsiveness in patients undergoing robot-assisted gynecologic surgery: a prospective observational study

**DOI:** 10.3389/fmed.2024.1387433

**Published:** 2024-04-04

**Authors:** Xixi Tang, Jingqiu Liang, Dongling Tan, Qi Chen, Chengfu Zhou, Tingjun Yang, Hongliang Liu

**Affiliations:** ^1^Department of Anesthesiology, Chongqing University Cancer Hospital, Chongqing, China; ^2^Chongqing Cancer Multi-Omics Big Data Application Engineering Research Center, Chongqing University Cancer Hospital, Chongqing, China; ^3^Department of Anesthesiology, People’s Hospital of Shizhu, Chongqing, China

**Keywords:** Doppler ultrasound, corrected flow time, fluid responsiveness, robotic-assisted, gynecologic surgery

## Abstract

**Background:**

The aim of this study was to evaluate the ability of point-of-care Doppler ultrasound measurements of carotid corrected flow time and its changes induced by volume expansion to predict fluid responsiveness in patients undergoing robot-assisted gynecological surgery.

**Methods:**

In this prospective study, carotid corrected flow time was measured using Doppler images of the common carotid artery before and after volume expansion. The stroke volume index at each time point was recorded using noninvasive cardiac output monitoring with MostCare. Of the 52 patients enrolled, 26 responded.

**Results:**

The areas under the receiver operating characteristic curves of the carotid corrected flow time and changes in carotid corrected flow time induced by volume expansion were 0.82 and 0.67, respectively. Their optimal cut-off values were 357 and 19.5 ms, respectively.

**Conclusion:**

Carotid corrected flow time was superior to changes in carotid corrected flow time induced by volume expansion for predicting fluid responsiveness in this population.

## 1 Introduction

Perioperative fluid management is essential to control vascular tone, ensure tissue perfusion, maintain the circulating volume, and improve cardiac output. Hypovolemia and hypervolemia can increase perioperative complications, including pulmonary edema, electrolyte imbalance, hemodilution coagulopathy, tissue hypoperfusion, and acid–base derangements. Optimizing perioperative fluid treatment often improves postoperative outcomes, reduces perioperative complications, and shortens hospital stays (1, 2). Therefore, assessing volume status and responsiveness is essential for fluid management in patients undergoing surgery and with critical illnesses. According to the Frank–Starling principle, increasing preload causes an increase in contractile strength and an increase in left ventricular stroke volume only if the ventricle is functioning on the steeply rising portion of the Frank–Starling curve. Fluid responsiveness can be used to identify and treat those patients who may benefit from an increase in venous volume through a fluid challenge to avoid volume overload (3, 4). Common metrics for assessing volume status and responsiveness include static (central venous, global end-diastolic volume index, and pulmonary arterial wedge pressure) and dynamic (stroke volume variation, pulse pressure variation) indicators. The static indicators used clinically cannot accurately assess volume status (5, 6), in contrast, dynamic indicators are derived from cardiopulmonary interactions (the passive leg raising test, end-expiratory occlusion test, and tidal volume challenge) during mechanical ventilation and may be helpful in guiding fluid management. Although functional hemodynamic parameters have been shown to reliably predict fluid responsiveness, factors such as pulmonary compliance, cardiac function, and mechanical ventilation may limit their broad clinical applications, including their application in certain surgical types and positions (7, 8). Therefore, an ideal hemodynamic monitoring technique should be less invasive, continuously dynamic, simple, generalized, and inexpensive to operate. We surmised that bedside ultrasound techniques that have emerged in recent years, such as the carotid corrected flow time (FTc), could be potential alternatives for predicting fluid volume in patients in the Trendelenburg, prone, or other position. Our group has proven that the FTc after tidal volume challenge reliably predicts fluid responsiveness in patients undergoing robot-assisted laparoscopic gynecological surgery in the modified head-down lithotomy position (9). In addition, the FTc measured in the common carotid artery is considered a reliable and efficient method to predict fluid reactivity (10–12). Previous studies have found that changes in FTc induced by the recruitment maneuver or the passive leg raise test could effectively identify “fluid responsive” patients (13, 14).

The primary aim of our research was to evaluate the value of point-of-care FTc and absolute changes in FTc (ΔFTc) induced by volume expansion (VE) in predicting fluid responsiveness among patients undergoing robot-assisted gynecological surgery.

## 2 Methods

### 2.1 Study population

After obtaining approval from the Institutional Review Board of Chongqing University Cancer Hospital (approval number: CZLS2021041-A, date of approval: April 1, 2022) and registering in the Chinese Clinical Trial Register (CHiCTR2200060573), this prospective study was conducted at Chongqing University Cancer Hospital between June and October 2022. This study protocol conformed to the tenets of the Declaration of Helsinki and has been reported in line with the Standards for the Reporting of Diagnostic accuracy studies (STARD) criteria (15). Written informed consent was obtained from all participants. We enrolled 55 patients scheduled to undergo robot-assisted gynecological surgery with American Society of Anesthesiologists (ASA) classes I–III. The exclusion criteria were a body mass index of more than 30 or less than 15 kg/m^2^, arrhythmia, decreased cardiac function (left ventricular ejection fraction less than 50%, right ventricular dysfunction), severe valve regurgitation, a history of carotid artery stenosis of 50%, chronic obstructive pulmonary disease, chronic kidney dysfunction, pregnancy, or denial to participate in the study.

### 2.2 Anesthesia technique

The patients were placed in the supine position in the operating room and subjected to standard monitoring using the IntelliVue MP40 monitor (Philips Medizin Systeme Boblingen GmbH, Boeblingen, Germany), for noninvasive blood pressure, heart rate (HR), continuous five-lead electrocardiography, and peripheral oxygen saturation. No preoperative medications were used in any patient. Anesthesia induction using propofol (2–3 mg/kg), sufentanil (0.3–0.5 μg/kg), midazolam (1–2 mg), and tracheal intubation was facilitated after 1 min of intravenous rocuronium (0.6–0.9 mg/kg) administration. Intraoperative anesthesia was maintained with continuous intravenous infusion of propofol (1.5–3 mg/kg/h), remifentanil (0.02–0.2 μg/kg/min), and sevoflurane (1–3 vol%). Neuromuscular blockade was maintained with rocuronium (0.15 mg/kg) administered at 30–40-min intervals. The radial artery was catheterized after the induction of anesthesia. Mechanical ventilation was controlled using a WATO EX-65 anesthesia machine (Mindray Medical Systems, Shenzhen, China), and the tidal volume was adjusted at 6 mL/kg of the predicted body weight in the volume-controlled mode. An end-tidal carbon dioxide concentration between 35 and 45 mmHg was maintained by controlling the respiratory rate, and the positive end-expiratory pressure was set at 5 cm H_2_O. At the beginning of surgery, the patients were placed in the modified head-down lithotomy position. Pneumoperitoneum was achieved by continuous carbon dioxide insufflation, and the intra-abdominal pressure was maintained at 12 mmHg. Intraoperative maintenance fluids were administered at a rate of 4 mL/kg per hour of Ringer’s solution.

### 2.3 Hemodynamic monitoring

Following arterial insertion, the arterial pressure signal was simultaneously connected and transmitted to the IntelliVue monitor and the hemodynamic monitoring system of the MostCare device (Vygon, Vytech, Padova, Italy) using a Y-cable. Each patient was positioned for surgery, and the arterial signal was calibrated to zero. MostCare, a multiparameter hemodynamic monitoring tool, utilizes pressure recording analysis methods to estimate cardiac output without requiring calibration. By analyzing the arterial waveform signal sampled at a high rate of 1,000 Hz, MostCare accurately identifies the dicrotic notch’s position and calculates the area under the arterial pressure waveform and systemic vascular impedance to determine the stroke volume (16, 17). Notably, the square-wave test was performed to ensure the normalcy of the arterial waveform after connecting the monitor, effectively excluding any under- or over-damping of the pressure signal (18).

### 2.4 Study procedures

The study was performed at least 45 min after the establishment of pneumoperitoneum, when the patients were hemodynamically stable [i.e., the change in the mean arterial pressure (MAP) and HR was less than 10% over 5 min] and the intra-abdominal pressure was maintained at 12 mmHg. In order to eliminate the potential effects of external interventions on peripheral vascular resistance and to improve data reliability, vasoactive medications were not administered during the study period. Upon program initiation, baseline hemodynamic variables including HR, MAP, pulse pressure variation (PPV), and stroke volume index (SVI) were obtained, and carotid FTc was measured (T0). Subsequently, a 250-mL infusion of Ringer’s solution was administered for 10 min. Hemodynamic variables were re-recorded 5 min after fluid expansion (T1). Fluid responsiveness was defined as an SVI increase of ≥10% after fluid administration, and the patients were classified as responders based on SVI ≥ 10% after the volume loading test, otherwise as non-responders (14).

### 2.5 Carotid ultrasonography

**Figure 1:**
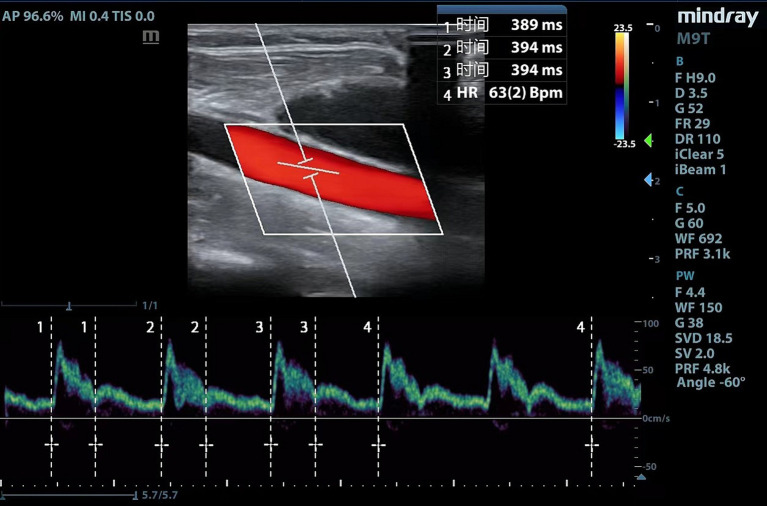
Carotid Doppler waveform.

Ultrasound examinations and measurements are performed by experienced physicians using a portable ultrasound equipment (Mindray Medical Systems, Shenzhen, China). Considering that part of the patients underwent right internal jugular vein cannulation, we uniformly measured left common carotid artery. The following scanning protocol was followed to acquire images of the common carotid artery: (1) a high-frequency line array transducer was placed transversely at the lower border of the thyroid cartilage, ensuring the common carotid artery was centered on the screen; (2) the long axis B-mode image of the common carotid artery was obtained with the probe marker pointing toward the patient’s head; (3) the sample volume was placed at the center of the arterial vessel, and the cursor angle was adjusted parallel to the direction of blood flow, with an insonation angle of ≤60°, approximately 2 cm proximal to the carotid bifurcation; (4) a satisfactory spectrum displayed was frozen by adjusting the optimal sampling volume and angle, and then, the measurement was performed using the caliper function on the machine (10, 13). Flow time (FT) was measured from the beginning of the systolic upstroke to the dicrotic notch. HR was automatically calculated from measurement intervals between the beginning of two consecutive Doppler flow upstrokes. The average of three consecutive cycles was recorded once stability was achieved and the quality reached an acceptable level (Figure 1). FTc was calculated for the compensation of HR using the Wodey formula: FTc = FT + [1.29 × (HR − 60)]. ΔFTc was calculated as follows: ΔFTc = FTc_T1_ − FTc_T0_ (19).

### 2.6 Sample size calculation and statistical analysis

PASS ver.15.0 (IBM Corp, Armonk, NY, United States) was used to calculate the sample size. According to Yang et al., the area under the curve (AUC) to predict fluid responsiveness was 0.82 in the descending aorta FTc. We hypothesized that the carotid FTc might have a low predictive capacity of 0.75 (12). We compared this value with the null hypothesis (AUC = 0.50, ratio of sample sizes in the negative/positive groups = 1) and generated a sample size of 50 patients (type I error = 0.05, power = 0.90). With an expected dropout rate of 10%, 55 patients were included in our study.

Normality was evaluated for all quantitative data using Kolmogorov–Smirnov test. Data are expressed as mean (standard deviation), median (interquartile range), and absolute number or percentage (%). Continuous variables of patient characteristics were compared between the groups using an independent-sample *t*-test or Mann–Whitney U test and the chi-squared test for categorical data. Hemodynamic parameters before and after VE were assessed using the paired *t*-test or Wilcoxon signed-rank sum test. In contrast, between-group comparisons were performed using the *t*-test or Mann–Whitney U test.

The ability of FTc and ΔFTc as predictors of fluid responsiveness was assessed using receiver operating characteristic (ROC) curve analysis, and different ROC curves were compared using the DeLong method (20). The optimal thresholds were defined using the maximum Youden index (sensitivity + specificity − 1) (21). We applied a gray-zone approach to describe an inconclusive range, considering threshold values corresponding to a sensitivity and specificity of 90% (22). Pearson correlation coefficient was used to investigate the association between carotid ultrasound variables and percentage changes in SVI after VE. The value of the correlation (*r*) coefficient ranges from −1 and 1; the closer the absolute value of *r* to 1, the stronger the correlation between the measured parameter and fluid responsiveness.

All statistical analyses were conducted using MedCalc ver. 20.1.0 (MedCalc Software, Ostend, Belgium), GraphPad Prism ver. 9.4.0 (GraphPad Software, San Diego, CA, United States), and SPSS ver. 27.0 (IBM Corp, Armonk, NY, United States). Results with *p* values <0.05 were regarded as statistically significant.

## 3 Results

**Figure 2:**
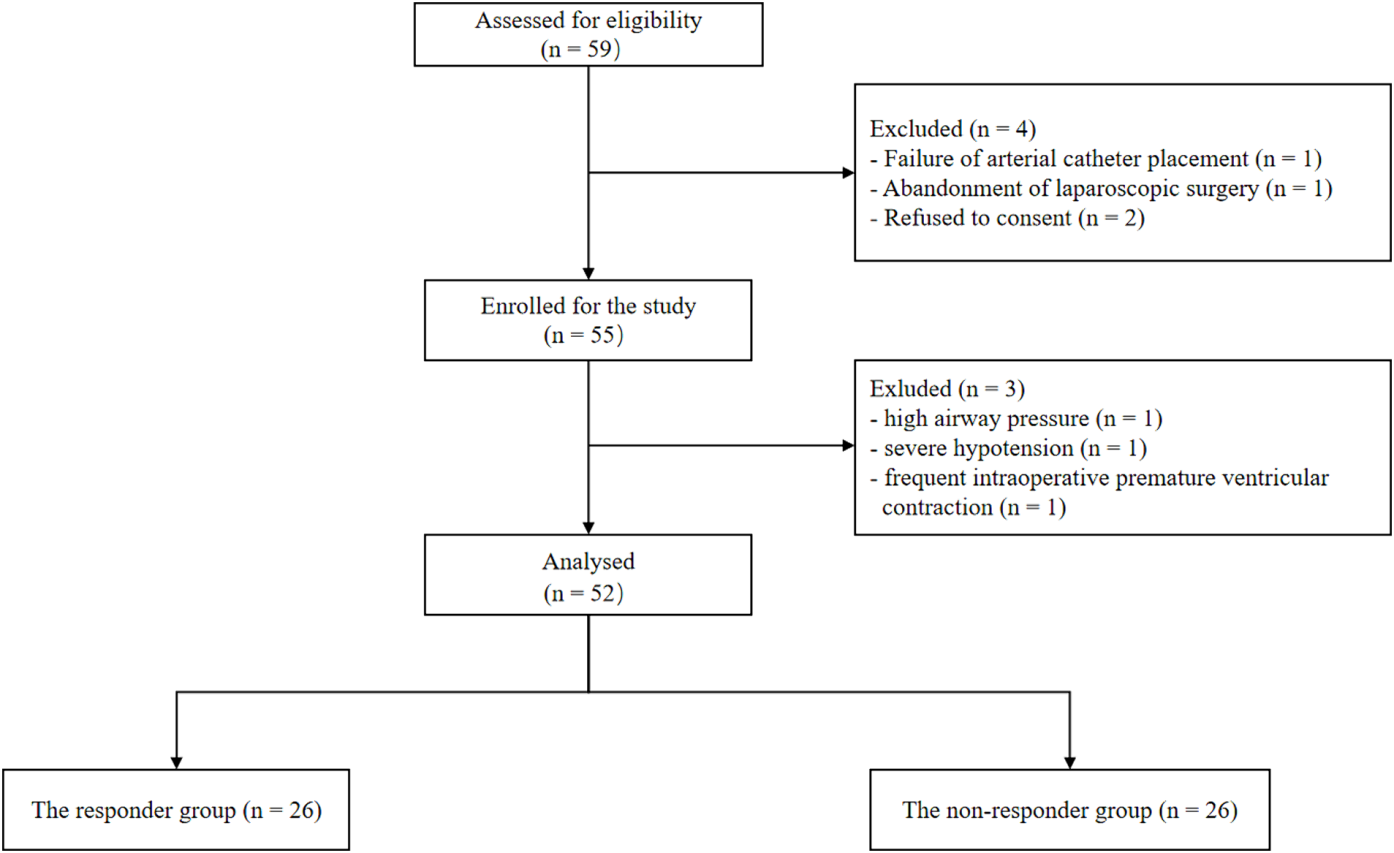
Flowchart of the study.

Of the 59 initially screened patients, 55 were enrolled in the study. Three patients were excluded because of excessive airway pressure, or unexpected cardiac arrhythmia and severe hypotension. Ultimately, 52 patients were analyzed in this study, and the number of responders and non-responders was the same (Figure 2). The demographics of the participants are shown in Table 1. No significant differences were observed in patient characteristics between the responders and non-responders.

**Table 1:** Patient characteristics.

	Overall (*n* = 52)	Responder (*n* = 26)	Non-responders (*n* = 26)	*p* value
Age, year	50.9 ± 10.9	51.4 ± 10.1	50.5 ± 11.8	0.773^a^
BMI, kg/m^2^	23.6 ± 3.2	23.6 ± 3.6	23.7 ± 2.8	0.966^a^
ASA physical status (II/III)	43,9	22,4	21,5	1^b^
Diagnosis, *n* (%)				0.637^c^
Vaginal cancer	6 (11.5)	4 (15.4)	2 (7.7)	
Cervical cancer	28 (53.8)	14 (53.8)	14 (53.8)	
Endometrial cancer	18 (34.6)	8 (30.8)	10 (38.5)	
Comorbidities, *n* (%)				0.206^c^
Hypertension	5 (9.6)	1 (3.8)	4 (13.8)	
Others	5 (9.6)	4 (13.8)	1 (3.8)	

BMI, Body mass index; ASA, American Society of Anesthesiologists physical status; ^a^Independent *t*-test, ^b^Mann–Whitney U, ^c^Chi-square test; Data are presented as mean (standard deviation), number of patients (%).

The hemodynamic, ventilatory, and ultrasound parameters before and after VE are presented in Table 2. A higher MAP was observed in the non-responders than in the responders at each time point, but there was no difference before and after rehydration. The baseline PPV, SVI, and FTc were comparable between the responders and non-responders, and PPV significantly decreased in both groups after VE, whereas the FTc significantly increased. In contrast, SVI after VE showed significant changes only in the responders.

**Table 2:** The hemodynamic, ventilatory, and ultrasound parameter characteristics before and after volume expansion.

	T0	T1	P (T0 vs. T1)
HR (beats/min)			
R	68.7 ± 9.5	66.9 ± 7.8	0.016
NR	65.6 ± 8.3	68.3 ± 7.3	0.002
Map (mm Hg)			
R	75.6 ± 6.7^*^	75.5 ± 6.4^*^	0.689
NR	80.3 ± 8.4	80.4 ± 8.5	0.889
Pplat (cm H₂O)			
R	20.9 ± 3.3	21.4 ± 3.1	0.004
NR	20.6 ± 2.8	21.0 ± 2.7	0.009
VT (mL)			
R	319.1 ± 31.4	318.2 ± 32.9	0.697
NR	323.5 ± 24.2	323.1 ± 23.3	0.777
Crs (mL/cmH₂O)			
R	17.0 ± 4.1	16.6 ± 3.8	0.11
NR	17.8 ± 3.2	17.2 ± 2.8	0.013
PPV (%)			
R	7.0 (4.8–9.0)^*^	6.0 (4.8–6.3)^*^	0.014
NR	5.0 (4.0–7.0)	4.0 (3.8–5.3)	0.003
SVI (mL/min^2^)			
R	41.4 ± 9.8^*^	46.5 ± 10.1	<0.001
NR	48.2 ± 6.3	48.2 ± 7.1	0.932
FTc (ms)			
R	347.3 ± 11.4^*^	364.5 ± 11.8^*^	<0.001
NR	362.7 ± 15.1	373.1 ± 17.2	<0.001

HR, Heart rate; MAP, Mean arterial pressure; Pplat, Plateau pressure; VT, Tidal volume; Crs, Respiratory compliance, PPV, Pulse pressure variation; SVI, Stroke volume index; FTc, Carotid corrected flow time; R, Responder; NR, Non-Responders. Data are mean ± standard deviation or median (interquartile range). ^*^p < 0.05 comparison between responders and non-responders at each time point.

**Figure 3:**
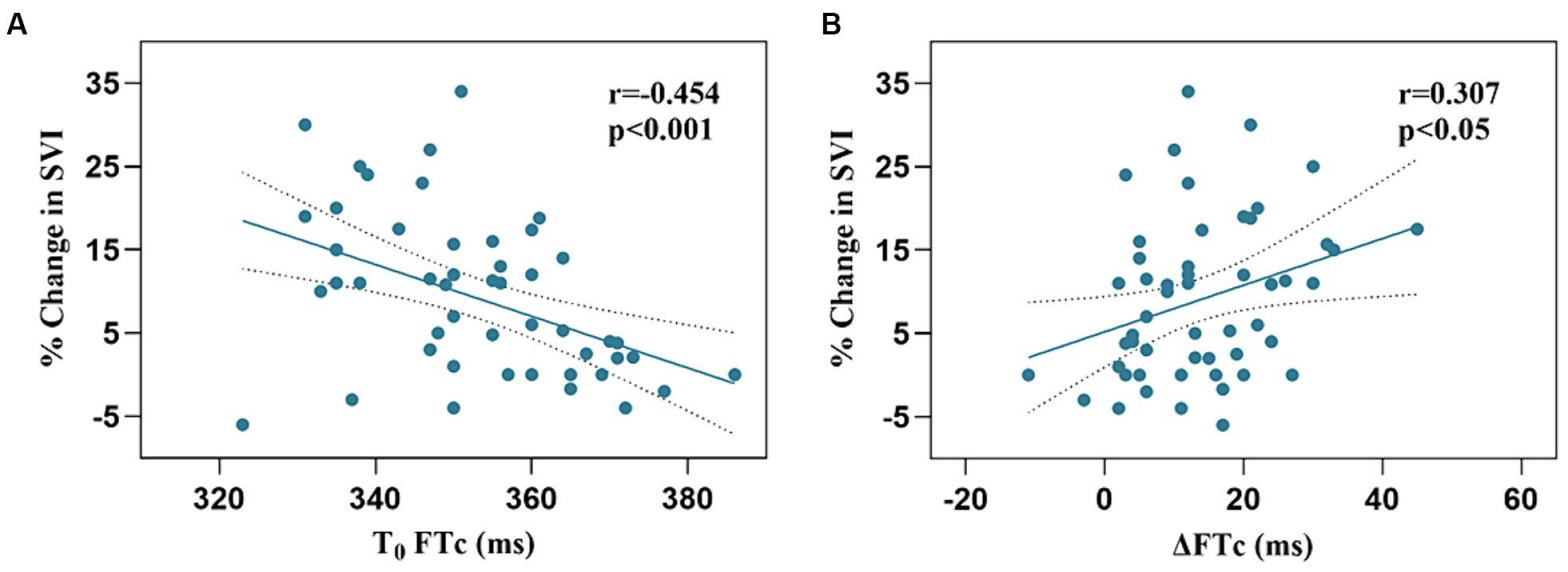
Relationship among FTc, ∆FTc, and SVI. A-SVI and FTC; B-SVI and ΔFTc.

The responders showed a more significant increase in FTc after VE than the non-responders (17.2 ± 10.9 vs. 10.4 ± 9.0 ms, *p* < 0.05). There was a negative correlation between baseline FTc and percentage change in SVI after VE (*r* = −0.454, 95% confidence interval [CI]: −0.647–0.207, *p* < 0.001), and a positive correlation between ΔFTc and percentage change in SVI after VE (*r* = 0.307, 95% CI: 0.371–0.535, *p* < 0.05) (Figure 3).

**Figure 4:**
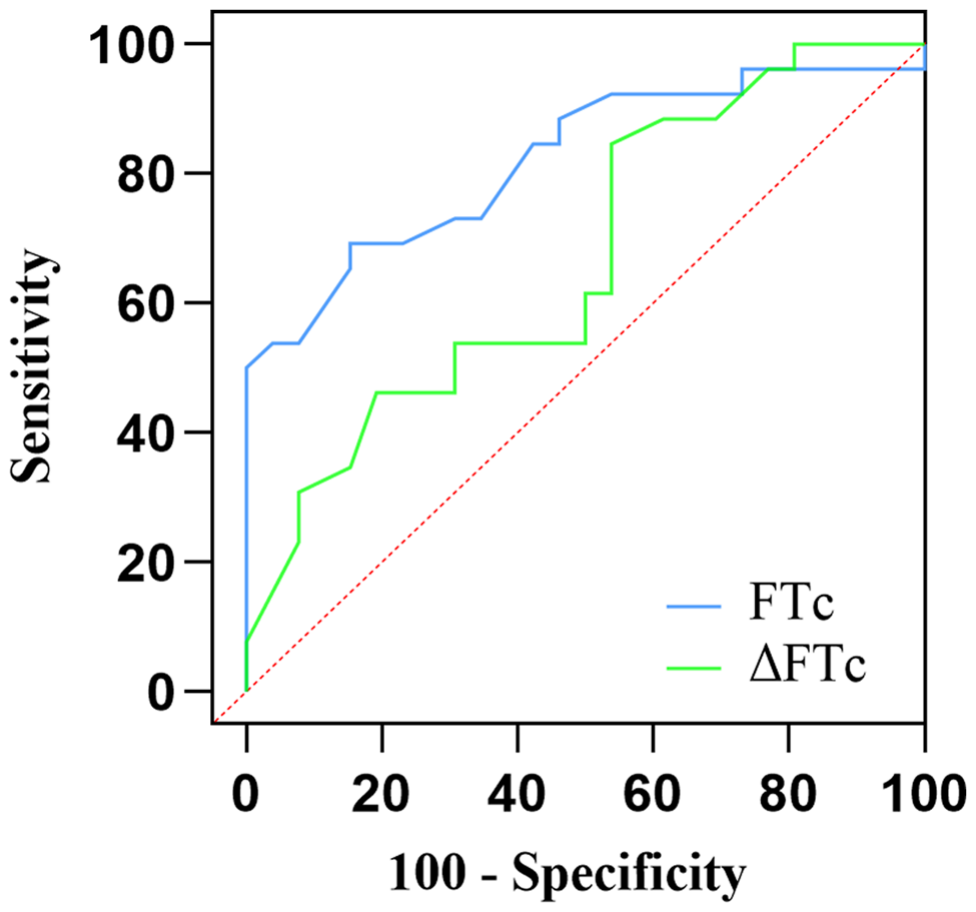
Comparison of the receiver operating characteristic curves for the prediction of fluid responsiveness.

The AUC value for FTc was 0.82 (95% CI: 0.705–0.937; *p* < 0.0001), showing excellent predictive capability for fluid responsiveness. The optimal cut-off value of FTc was 357 ms, with a sensitivity and specificity of 69.2 and 84.6%, respectively. In contrast, the ability of ΔFTc to predict fluid responsiveness with an AUC of 0.67 (95% CI: 0.520–0.815; *p* < 0.05) showed a lower accuracy (Figure 4). The optimal cut-off value of ΔFTc was 19.5 ms, with a sensitivity and specificity of 84.6 and 46.2%, respectively. However, for the predictive accuracy of fluid responsiveness, there was no significant difference in the AUC between the FTc and ΔFTc groups (*p* = 0.12). The gray zone for FTc (347.1–359.9 ms) contained 14 patients (27%). The gray zone for ΔFTc (4.7–22.2 ms) contained 33 (63%) patients (Table 3).

**Table 3:** Diagnostic ability of different parameters to predict fluid responsiveness.

	AUC (95%CI)	*p* value	Cut of value	Sensitivity (95%CI)	Specificity (95%CI)	Gray zone	Patients in gray zone (%)
FTc	0.82 (0.705*–*0.937)	<0.0001	357 ms	69.2 (50.0*–*83.5)	84.6 (66.5*–*93.9)	347.1*–*359.9 ms	27
ΔFTc	0.67 (0.520*–*0.815)	<0.05	19.5 ms	84.6 (66.5*–*93.9)	46.2 (28.8*–*64.5)	4.7*–*22.2 ms	63

FTc, Carotid corrected flow time; ΔFTc, Changes in the corrected carotid flow time; AUC, Area under the receiver operating characteristic curve; and CI, Confidence interval.

## 4 Discussion

Among gynecologic patients undergoing robot-assisted laparoscopic surgery in the modified head-down lithotomy position, based on the results of our study, the carotid FTc assessed using Doppler ultrasound is an excellent predictor of fluid responsiveness. Although ΔFTc induced by VE also possessed the ability to predict fluid responsiveness, it showed only moderate capability. Compared to FTc, the broader gray area for ΔFTc, containing 63% of the patients, potentially limits its clinical effectiveness.

Perioperative fluid management is directly related to patient survival, and volume assessment is essential for guiding the individualization and accuracy of intraoperative fluid therapy. Monitoring has evolved from being static to dynamic. In recent years, researchers have focused on developing noninvasive techniques with high accuracy and precision, thus avoiding the complications of invasive monitoring and analyzing the response to fluid therapy (5–8, 23). Portable ultrasound has been widely recommended for volume status assessment in critical care, emergency, and perioperative patients because of its convenient, noninvasive, easy-to-acquire, and reproducible characteristics.

Recent studies have increasingly identified a significant correlation between FTc and changes in intravascular volume status (10, 11, 24). Accordingly, as intravascular volume decreases, FTc tends to decrease. In contrast, volume-deficient patients experience an increase in FTc following volume infusion. Patients with advanced renal failure show significantly lower FTc values after hemodialysis, and there is a significant negative correlation between the volume of fluid excreted during hemodialysis and changes in FTc (11). Mackenzie et al. (24) found that FTc decreases after acute blood loss and that passive leg raising restores FTc to predicted levels in acute hypovolemia. Assessing the correlation between the changes in volume status and those in carotid FTc, Blehar et al. reported that dehydrated patients receiving fluid resuscitation showed an increase in carotid FTc from a mean of 299 ms before injection to a mean of 340 ms after injection. Moreover, the carotid FTc responds more significantly to changes in intravascular volume than to negligible changes in HR and MAP (10). Similarly, our results showed an increase in FTc in both groups of patients after fluid injection, a more pronounced increase in the responders, and no significant changes in blood pressure.

A growing body of evidence highlights the significance of ultrasound measurement of carotid FTc in volume management. Kim et al. (12) identified that FTc could accurately predict volume responsiveness in spontaneously breathing patients with an AUC of 0.84. Jung et al. considered carotid FTc as a reliable predictor to assess fluid responsiveness in patients with low tidal volume mechanical ventilation. A systematic review revealed that the diagnostic characteristics of FTc varied, with the sensitivity ranging from 60 to 73%, specificity from 82 to 92%, and optimal cutoff of AUC from 0.7526 to 0.8819 (25). A higher optimal cutoff value of 357 ms obtained in our study could be attributed to the patient’s positioning, potentially increasing venous return and affecting the FTc measurement values.

The absolute value of FTc alone as a static indicator has limitations as it depends on left ventricular preload, cardiac inotropy, and systemic vascular resistance (26). Based on the above findings, researchers have hypothesized that the changes in FTc could be considered an indicator of fluid reactivity. Jalil et al. first attempted to determine whether passive leg raising-induced increase in FTc could be used to predict fluid responsiveness in critically ill patients with a cardiac output monitor, ultimately concluding that an increase of ≥24.6% in the FTc in response to passive leg raising is a reasonable predictor of fluid responsiveness (27). In patients with early undifferentiated shock, Barjaktarevic et al. demonstrated, through prospective experiments, that not only the changes in FTc evoked by a passive leg raising operation can determine fluid responsiveness, but also a threshold of 7 ms as FTc to define fluid responsiveness (sensitivity of 68% and specificity of 96%) (14). Passive leg raising involves elevation of the lower extremities in order to rapidly divert venous blood and ultimately increase return blood volume. While a straightforward method of assessing volume status, it is associated with the risk of causing an increase in intracranial pressure and a decrease in pulmonary compliance, and its implementation during surgery is challenging (28). Consequently, alternative interventions are necessary. A subsequent experiment directly illustrated that the percent change in FTc induced by the recruitment maneuver used to predict fluid responsiveness in supine patients under general anesthesia is feasible (13). In our study, we obtained a higher absolute value for ΔFTc with a cutoff value of 19.5 ms (sensitivity of 84.6% and specificity of 46.2%). Regardless of whether the threshold for a change in FTc with a fluid challenge varied from 7 to 30 ms or a 25% relative change, possible reasons for this difference include the following. First, our participants were in a head-down position, which would have increased venous return and cardiac preload. Second, the hemodynamic effects of pneumoperitoneum were complex. Finally, we identified responders and non-responders by additional fluid supplementation (29, 30). Therefore, it can be concluded that the ΔFTc increases significantly in patients who respond to fluids after intravenous infusion or passive leg raising.

This study had several limitations. We did not utilize the gold standard thermodilution method for monitoring cardiac output; instead, we used a less invasive continuous hemodynamic monitor. Although the MostCare monitor showed good agreement with echocardiographic measurements, potential errors may be unavoidable under the dual effects of pneumoperitoneum and head-down position. The effects of cerebrovascular tension on the common carotid artery cannot be excluded entirely, and brain autoregulatory mechanisms and blood carbon dioxide levels correlate with the former. Finally, the results obtained cannot be completely generalized to other patients because of the specificity of the type of disease and the surgical approach, such as facial and cerebral surgery or other open surgery.

In conclusion, carotid parameters were measured using Doppler ultrasound in gynecologic patients undergoing robotic-assisted laparoscopic surgery, and FTc seems to be a more reasonable predictor than ΔFTc induced by VE. We will continue to conduct additional clinical trials and offer additional reference points for perioperative volume management.

## Data availability statement

The original contributions presented in the study are included in the article/supplementary material; further inquiries can be directed to the corresponding authors.

## Ethics statement

The studies involving humans were approved by Institutional Review Board of Chongqing University Cancer Hospital (approval number: CZLS2021041-A, date of approval: April 1, 2022). The studies were conducted in accordance with the local legislation and institutional requirements. Written informed consent for participation in this study was provided by the participants’ legal guardians/next of kin.

## Author contributions

XT: Writing – original draft. JL: Writing – original draft, Funding acquisition, Supervision. DT: Writing – original draft, Formal analysis. QC: Formal analysis, Writing – original draft, Data curation, Resources, Software. CZ: Resources, Supervision, Validation, Writing – original draft. TY: Conceptualization, Writing – original draft. HL: Conceptualization, Writing – review & editing.

## References

[1] Fantoni D Shih AC. Perioperative fluid therapy. Vet Clin North Am Small Anim Pract. (2017) 47:423–34. doi: 10.1016/j.cvsm.2016.11.004 28164837

[2] Wellge BE Trepte CJ Zöllner C Izbicki JR Bockhorn M. Perioperatives volume management [perioperative fluid management]. Chirurg. (2020) 91:121–7. doi: 10.1007/s00104-020-01134-6, PMID: 32025774

[3] Cecconi M Parsons AK Rhodes A. What is a fluid challenge? Curr Opin Crit Care. (2011) 17:290–5. doi: 10.1097/MCC.0b013e32834699cd 21508838

[4] Myatra SN Monnet X Teboul JL. Use of 'tidal volume challenge' to improve the reliability of pulse pressure variation. Crit Care. (2017) 21:60. doi: 10.1186/s13054-017-1637-x, PMID: 28320434 PMC5359814

[5] Marik PE Cavallazzi R. Does the central venous pressure predict fluid responsiveness? An updated meta-analysis and a plea for some common sense. Crit Care Med. (2013) 41:1774–81. doi: 10.1097/CCM.0b013e31828a25fd, PMID: 23774337

[6] Osman D Ridel C Ray P Monnet X Anguel N Richard C. Cardiac filling pressures are not appropriate to predict hemodynamic response to volume challenge. Crit Care Med. (2007) 35:64–8. doi: 10.1097/01.CCM.0000249851.94101.4F 17080001

[7] Hofer CK Cannesson M. Monitoring fluid responsiveness. Acta Anaesthesiol Taiwanica. (2011) 49:59–65. doi: 10.1016/j.aat.2011.05.001 21729812

[8] Monnet X Marik PE Teboul JL. Prediction of fluid responsiveness: an update. Ann Intensive Care. (2016) 6:111. doi: 10.1186/s13613-016-0216-7, PMID: 27858374 PMC5114218

[9] Tang X Chen Q Huang Z Liang J An R Liu H. Comparison of the carotid corrected flow time and tidal volume challenge for assessing fluid responsiveness in robot-assisted laparoscopic surgery. J Robot Surg. (2023) 17:2763–72. doi: 10.1007/s11701-023-01710-y, PMID: 37707743

[10] Blehar DJ Glazier S Gaspari RJ. Correlation of corrected flow time in the carotid artery with changes in intravascular volume status. J Crit Care. (2014) 29:486–8. doi: 10.1016/j.jcrc.2014.03.025, PMID: 24930363

[11] Hossein-Nejad H Mohammadinejad P Lessan-Pezeshki M Davarani SS Banaie M. Carotid artery corrected flow time measurement via bedside ultrasonography in monitoring volume status. J Crit Care. (2015) 30:1199–203. doi: 10.1016/j.jcrc.2015.08.014, PMID: 26410681

[12] Kim DH Shin S Kim N Choi T Choi SH Choi YS. Carotid ultrasound measurements for assessing fluid responsiveness in spontaneously breathing patients: corrected flow time and respirophasic variation in blood flow peak velocity. Br J Anaesth. (2018) 121:541–9. doi: 10.1016/j.bja.2017.12.047, PMID: 30115251

[13] Kimura A Suehiro K Juri T Tanaka K Mori T. Changes in corrected carotid flow time induced by recruitment maneuver predict fluid responsiveness in patients undergoing general anesthesia. J Clin Monit Comput. (2022) 36:1069–77. doi: 10.1007/s10877-021-00736-7, PMID: 34191254

[14] Barjaktarevic I Toppen WE Hu S Aquije Montoya E Ong S Buhr R. Ultrasound assessment of the change in carotid corrected flow time in fluid responsiveness in undifferentiated shock. Crit Care Med. (2018) 46:e1040–6. doi: 10.1097/CCM.0000000000003356, PMID: 30134304 PMC6774608

[15] Bossuyt PM Reitsma JB Bruns DE Gatsonis CA Glasziou PP Irwig L. STARD 2015: an updated list of essential items for reporting diagnostic accuracy studies. BMJ. (2015) 351:h5527. doi: 10.1136/bmj.h5527, PMID: 26511519 PMC4623764

[16] Critchley LA. Validation of the MostCare pulse contour cardiac output monitor: beyond the bland and Altman plot. Anesth Analg. (2011) 113:1292–4. doi: 10.1213/ANE.0b013e31822d6785, PMID: 22116965

[17] Romagnoli S Ricci Z Romano SM Dimizio F Bonicolini E Quattrone D. FloTrac/Vigileo(TM) (third generation) and MostCare(®)/PRAM versus echocardiography for cardiac output estimation in vascular surgery. J Cardiothorac Vasc Anesth. (2013) 27:1114–21. doi: 10.1053/j.jvca.2013.04.017, PMID: 24055563

[18] Messina A Montagnini C Cammarota G Giuliani F Muratore L Baggiani M. Assessment of fluid responsiveness in prone neurosurgical patients undergoing protective ventilation: role of dynamic indices, tidal volume challenge, and end-expiratory occlusion test. Anesth Analg. (2020) 130:752–61. doi: 10.1213/ANE.0000000000004494, PMID: 31651455

[19] Mohammadinejad P Hossein-Nejad H. Calculation of corrected flow time: Wodey’s formula vs. J Crit Care. (2018) 44:154–5. doi: 10.1016/j.jcrc.2017.10.046, PMID: 29127840

[20] DeLong ER DeLong DM Clarke-Pearson DL. Comparing the areas under two or more correlated receiver operating characteristic curves: a nonparametric approach. Biometrics. (1988) 44:837–45. doi: 10.2307/2531595 3203132

[21] Youden WJ. Index for rating diagnostic tests. Cancer. (1950) 3:32–5. doi: 10.1002/1097-0142(1950)3:1<32::aid-cncr2820030106>3.0.co;2-3 15405679

[22] Coste J Pouchot J. A gray zone for quantitative diagnostic and screening tests. Int J Epidemiol. (2003) 32:304–13. doi: 10.1093/ije/dyg054 12714554

[23] Scheeren TWL Ramsay MAE. New developments in hemodynamic monitoring. J Cardiothorac Vasc Anesth. (2019) 33:S67–72. doi: 10.1053/j.jvca.2019.03.043 31279355

[24] Mackenzie DC Khan NA Blehar D Glazier S Chang Y Stowell CP. Carotid flow time changes with volume status in acute blood loss. Ann Emerg Med. (2015) 66:277–282.e1. doi: 10.1016/j.annemergmed.2015.04.014, PMID: 26003002

[25] Beier L Davis J Esener D Grant C Fields JM. Carotid ultrasound to predict fluid responsiveness: a systematic review. J Ultrasound Med. (2020) 39:1965–76. doi: 10.1002/jum.15301 32314817

[26] SINGER M ALLEN MJ WEBB AR BENNETT ED. Effects of alterations in left ventricular filling, contractility, and systemic vascular resistance on the ascending aortic blood velocity waveform of normal subjects. Crit Care Med. (1991) 19:1138–45. doi: 10.1097/00003246-199109000-00008, PMID: 1679385

[27] Jalil B Thompson P Cavallazzi R Marik P Mann J el-Kersh K. Comparing changes in carotid flow time and stroke volume induced by passive leg raising. Am J Med Sci. (2018) 355:168–73. doi: 10.1016/j.amjms.2017.09.006, PMID: 29406045

[28] Corl K Napoli AM Gardiner F. Bedside sonographic measurement of the inferior vena cava caval index is a poor predictor of fluid responsiveness in emergency department patients. Emerg Med Australas. (2012) 24:534–9. doi: 10.1111/j.1742-6723.2012.01596.x, PMID: 23039295

[29] Zeuzem-Lampert C Groene P Brummer V Hofmann-Kiefer K. Kardiorespiratorische Effekte perioperativer Positionierungsmaßnahmen [cardiorespiratory effects of perioperative positioning techniques]. Anaesthesist. (2019) 68:805–13. doi: 10.1007/s00101-019-00674-9, PMID: 31713665

[30] Antiperovitch P Iliescu E Chan B. Carotid systolic flow time with passive leg raise correlates with fluid status changes in patients undergoing dialysis. J Crit Care. (2017) 39:83–6. doi: 10.1016/j.jcrc.2017.02.017, PMID: 28231519

